# Research note: The chicken gut virome: Spatiotemporal dynamics and divergent responses to antibiotic versus phytogenic supplementation

**DOI:** 10.1016/j.psj.2026.106373

**Published:** 2026-01-03

**Authors:** Li Yang, Jinlong Ru, Shun Guo, Xueqin Yang, Pengying Li, Li Deng, Xia Wang

**Affiliations:** a College of Animal Science and Technology, Northwest A&F University, Yangling 712100 Shaanxi, China; b Technical University of Munich, Prevention of Microbial Diseases, TUM School of Life Sciences; Central Institute of Infection Prevention (ZIP), Freising 85354, Germany; c Research unit of Microbial Diseases Prevention, Institute of Virology, Helmholtz Centre Munich - German Research Centre for Environmental Health, Neuherberg 85764, Germany; d College of Animal Sciences, Xinjiang Agricultural University, Urumqi 830052, China; e College of Landscape Architecture and Art, Northwest A&F University, Yangling 712100, China

**Keywords:** Gut virome, Phytogenics, Feed additives, Antibiotic alternative, Phage-bacteria interaction

## Abstract

This study employed metagenomic sequencing data to comprehensively investigate the gut virome, with a focus on the bacteriophage communities (the phageome), across intestinal regions and developmental stages in 360 chickens. We characterized the spatiotemporal dynamics of phage communities and assessed the impact of chlortetracycline (CTC), an antibiotic, and *Macleaya cordata* extract (MCE), a phytogenic supplement. Our analysis revealed that phage community assembly was highly structured, exhibiting distinct successional patterns across age and between foregut and hindgut segments. A key finding was the identification of a potential antibiotic-phage synergy, mediated by phage-encoded auxiliary metabolic genes (AMGs) involved in bacterial immune evasion, suggesting a novel mechanism for enhanced infectivity under antibiotic pressure. In contrast, phytogenic supplementation promoted gut ecosystem homeostasis by fostering significantly richer and more diverse phage communities. Our results delineate the fundamental ecology of the chicken gut virome and provide mechanistic insights into how different growth promoters exert contrasting effects on viral populations, supporting the use of phytogenics as sustainable alternatives for animal husbandry.

## Introduction

The gastrointestinal tract (GIT) of poultry is a complex ecosystem harboring a diverse community of microorganisms, including bacteria, archaea, viruses, and fungi, which play a crucial role in host health, nutrient digestion, and immune system development. Among these microbial inhabitants, bacteriophages (or phages), viruses that specifically infect bacteria, are the most abundant biological entities. As key players in the gut microbiome, phages drive bacterial community dynamics through predator-prey relationships and horizontal gene transfer, influencing overall microbial diversity and stability (Naureen et al., 2020).

While the bacterial component of the chicken gut microbiome has been extensively studied, the viral fraction, particularly the phage community, remains relatively unexplored. A critical gap in our understanding lies in the temporal and spatial distribution of these phages along the digestive tract. The chicken GIT presents distinct physicochemical and biological environments from the crop to the ceca, which likely shape unique phage populations in each compartment (Bindari and Gerber, 2022). Furthermore, how these viral communities establish and evolve as the chicken matures is not well-characterized. Elucidating this spatial variation and temporal succession is fundamental to understanding the intricate phage-bacteria interactions that underpin gut homeostasis.

The balance of this gut ecosystem is highly susceptible to external modulators, with dietary and pharmaceutical interventions being primary factors. Antibiotics, historically used as growth promoters and for disease prevention in poultry farming, are known to cause profound disruptions in the gut bacterial microbiota (Hasan and Yang, 2019). However, their specific impact on the phageome remains largely unexplored. This represents a critical knowledge gap, as antibiotic-induced stress on bacteria is known to trigger profound shifts in phage life cycles (lytic vs. lysogenic). Such shifts can not only rapidly destabilize the viral community structure, but may also accelerate the dissemination of antibiotic resistance genes (Torres-Barceló, 2018). Given the global push to reduce antibiotic use in animal agriculture, phytogenics or Chinese herbal medicines have emerged as promising natural alternatives. These compounds are believed to possess antimicrobial, anti-inflammatory, and immunity-enhancing properties (Wang et al., 2024). Their influence on the gut bacterial community is an active area of research, but their specific effects on the phage community are virtually unknown. It is plausible that these herbal compounds selectively inhibit certain bacterial hosts, thereby indirectly shaping the phage populations that prey upon them.

Therefore, this study aims to comprehensively investigate the phage community in the intestines of chickens of different ages. We will first define the spatial and temporal dynamics of the phage community in different intestinal segments. Subsequently, we will evaluate the comparative impact of antibiotic administration and Chinese herbal extract supplementation on the structure and composition of this viral community. By deciphering the interactions between these modulators and the gut phageome, this research seeks to provide novel insights into managing gut health in poultry production, potentially leading to strategies for manipulating the phageome to foster a more robust and resilient microbial ecosystem.

## Materials and methods

### Metagenomic data source and selection

This study utilized publicly available metagenomic sequencing data from the chicken intestinal microbiome project PRJNA417359. Original animal procedures were approved as detailed in (Huang et al., 2018). The original data were generated using Illumina HiSeq 2500 and HiSeq X Ten platforms with a paired-end 150 bp (PE150) sequencing strategy, yielding an average of 12 million reads per sample. From the original dataset (n=495), a subset of 360 metagenomic samples was selected for analysis based on two stringent criteria: 1) completeness of metadata across all key experimental variables (developmental stage, intestinal segment, and treatment group) to enable robust multi-factorial comparisons; and 2) detectability of viral signals, as samples failing to yield identifiable viral contigs or sufficient viral reads during the initial screening were excluded to ensure analytical reliability. The selected samples were derived from Arbor Acres broilers (AA, n=180) and Local yellow-feather chickens (LY, n=180), both breeds having comprehensive spatiotemporal data.

### Experimental design and animal groups

The experimental design encompassed multiple age points and dietary treatments. AA broilers were sampled at five ages (1, 7, 14, 28, and 42 days), while LY chickens included an additional 56-day time point. At each age (except for day 1), chickens were randomly assigned to one of five dietary treatment groups. (1) BLANK: basal diet; (2) CTC: basal diet supplemented with 50 mg/kg Citifac® (chlortetracycline 20% w/w premix); (3) MCE-L, MCE-M, MCE-H: basal diet with 15, 50, 150 mg/kg Sangrovit® (*Macleaya cordata* extract 3.75% w/w premix), and in these groups' names, L stands for low, M for medium, and H for high. Only the BLANK group was included for the 1-day-old chicks.

### Metagenomic virome analysis: phage identification, classification, lifestyle prediction and host relationship study and AMG identification

To profile the gut virome, we employed our recently developed ViroProfiler pipeline (Ru et al., 2023), which integrates the key analytical stages from viral contig identification and quality control to taxonomic classification, functional annotation, and viral abundance estimation. Briefly, assembled contigs were initially screened with geNomad (version 1.8.0, –min-score 0.75). A refined identification was then performed: CheckV (version 1.0.1) removed host contamination and assessed completeness, and a consensus of VirSorter2 (version 2.2.3) and VIBRANT (version 1.2.1) results was used to retain viral contigs supported by at least two tools for downstream analysis. Putative bacterial hosts were predicted using iPHoP (version 1.3.3), with a high-confidence score threshold (score ≥ 90). For functional characterization, AMGs were identified and annotated using VIBRANT (version 1.2.1) and DRAM-v (version 1.3) with their default settings. Quality-filtered reads were aligned to the non-redundant viral catalog using Bowtie2, and viral abundance was quantified using CoverM (version 0.7.0).

### Phage composition analysis

For microbial diversity analysis, ACE, Chao1, Pielou's evenness, Observed Richness, Shannon, and Simpson indices were used. The overall differences in phage community structures were evaluated by non-metric multidimensional scaling (NMDS) based on Bray-Curtis dissimilarity values.

### Statistical analyses

All statistical analyses were performed in R (v4.4.2). Data manipulation and visualization utilized the tidyverse ecosystem (v1.3.0). Student’s t-test and Wilcoxon rank sum test were employed for group comparisons.

## Results and discussion

### The spatiotemporal heterogeneity of phage distribution in the gut

**Fig. 1:**
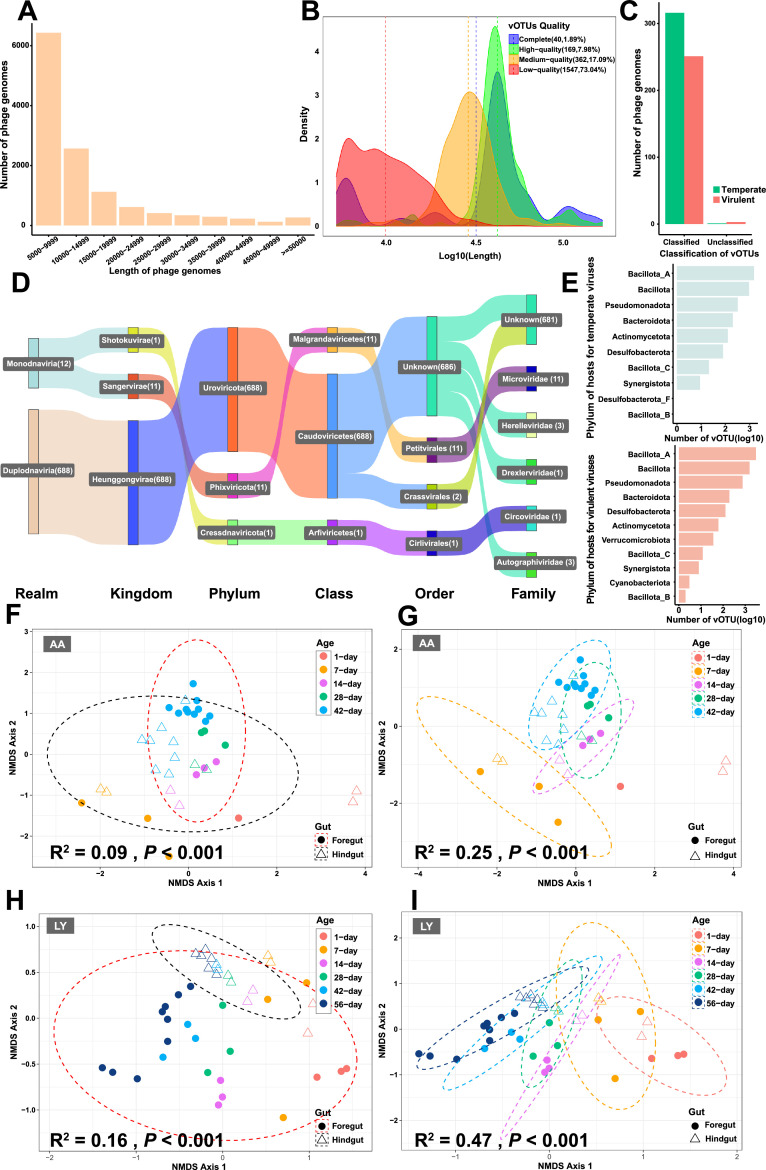
Characterization and spatiotemporal dynamics of the chicken gut phageome.

Metagenomic analysis of 360 chickens yielded 12,400 viral contigs, which were clustered into 2,118 viral operational taxonomic units (vOTUs) after quality filtering (571 of medium-to-high quality; Fig. 1A, B). Taxonomic classification assigned the majority to the classes Caudoviricetes, Malgrandaviricetes, and Arfiviricetes (Fig. 1C, D). Host prediction via iPHoP revealed 9,061 virus-host linkages, with lytic phages (5,831 vOTUs) showed a strong preference for Bacillota_A, while temperate phages (3,230 vOTUs) showed a broader host range across 10-11 bacterial phyla (Fig. 1E).

(A) Length distribution of the assembled gut phage genomes. (B) Quality assessment of vOTUs, categorized as complete (n=54), high-quality (n=193), medium-quality (n=453), or low-quality (n=2,688). The plot shows the density and count for each category. (C) Classification of vOTUs based on lifestyle (temperate vs. virulent). (D) The gut virological classification hierarchy Sankey diagram shows the distribution and quantitative relationships of classification levels from domain, phylum, class, order, to family. (E) Bar plot showing the number of temperate and virulent vOTUs linked to bacterial hosts at the phylum level. (F-I) NMDS plots of viral communities based on Bray-Curtis dissimilarity, displaying confidence ellipses. (F, H) NMDS ordination grouped by intestinal segment (Foregut vs. Hindgut) for AA broilers (F) and LY chickens (H). (G, I) NMDS ordination grouped by age for AA broilers (G) and LY chickens (I).

Analysis of the sample groups revealed significant spatiotemporal dynamics in the phage communities. NMDS showed clear separation between foregut and hindgut communities (AA: *R²* = 0.09, P < 0.001; LY: *R²* = 0.16, P < 0.001; Fig. 1F, H) and across developmental stages (AA: *R²* = 0.25, P < 0.001; LY: *R²* = 0.47, P < 0.001; Fig. 1G, I). Notably, the most substantial community shift occurred between 1 and 7 days of age. Furthermore, the primary site of age-related succession differed by breed: the hindgut was more dynamic in AA broilers, whereas the foregut changed more markedly in LY chickens.

Our results establish that the chicken gut virobiota is organized along two fundamental ecological gradients: space (intestinal segment) and time (age), which were consistent with the previous studies in mice, indicating that the intestinal viral compartment has a strong compartmentalization feature (Kim and Bae, 2016). The significant segregation between foregut and hindgut communities (Bray-Curtis distance; P < 0.001) likely originates from the distinct physiological environments of the small intestine (e.g., duodenum, jejunum) and the large intestine (e.g., cecum, colorectum). The small intestine is primarily responsible for the rapid digestion and absorption of nutrients, characterized by a relatively oxygen-rich, lower pH environment with a constant flux of nutrients. In contrast, the large intestine, particularly the cecum, functions as an anaerobic fermentation chamber with a near-neutral pH, rich in complex dietary fibers. This creates a unique ecological niche for strict anaerobes and their associated phages. The functional gradient from "digestion and absorption" to "fermentation" shapes distinct bacterial host communities, which in turn governs the structure of the phage communities (Zhao et al., 2025). Furthermore, the successional patterns of these phage communities were breed-specific. In AA broilers, optimized for rapid growth, the most dramatic changes occurred in the hindgut, the major site of fermentation and energy harvest. Conversely, in LY chickens, the foregut exhibited greater dynamism. This breed-level divergence may reflect genetic differences in niche adaptation along the intestinal tract. Variations in foregut physiology (e.g., enzyme secretion, motility) between LY and AA chickens could alter the ecological dynamics of both the microbiota and its phages. This implies that host genetics indirectly sculpt the virome by fine-tuning the physiological landscape of the gut. The most pronounced shift across all groups happened between day 1 and day 7, a period of intense post-hatching development where digestive enzyme activity surges and intestinal villi mature. This synchrony indicates that phage community assembly is an integral component of host gut development, rather than a passive process. The initial colonization and succession of phages appear to be orchestrated by the changing landscape of the gut itself, much like the ecological succession seen in a newly formed habitat.

### Differential effects of antibiotic and herbal additives on phage communities

**Fig. 2:**
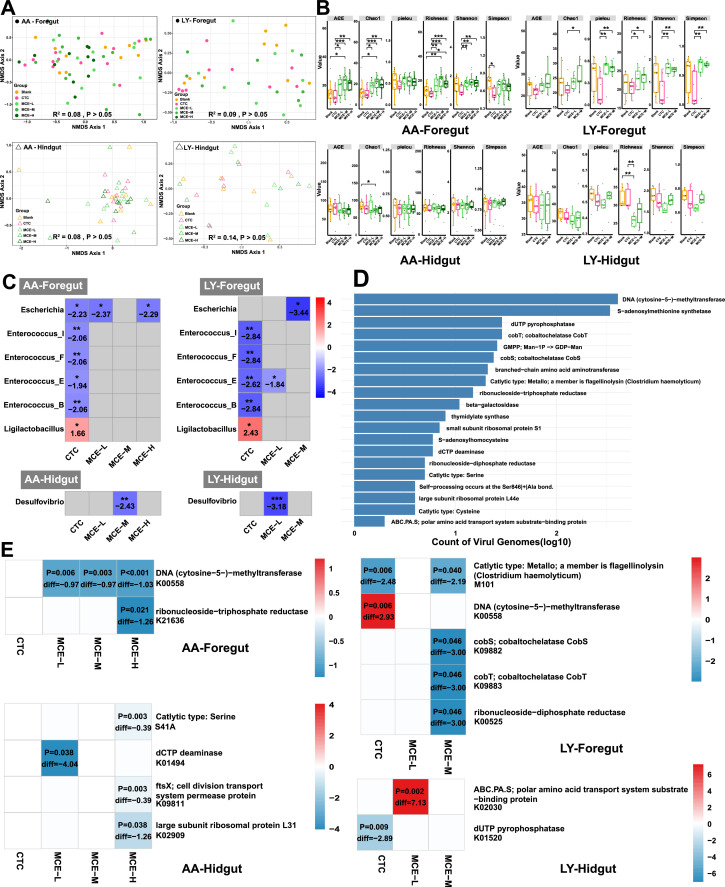
Modulation of gut phage communities by antibiotic and herbal additives.

Having established the baseline spatiotemporal dynamics, we investigated the impact of the CTC and the MCE on the gut phage communities. NMDS revealed no significant overall clustering among the Blank, CTC, and MCE groups (P > 0.05; Fig. 2A). However, alpha diversity analysis uncovered distinct, segment-specific responses (Fig. 2B).

(A) NMDS plot of viral communities from the foregut and hindgut of AA and LY chickens across all five treatment groups, based on Bray-Curtis dissimilarity. (B) Alpha diversity of the phage communities in the foregut and hindgut of AA and LY chickens under different treatments. Box plots display multiple diversity indices, including richness (Chao1, ACE, Observed OTUs) and diversity (Shannon, Simpson, Pielou). (C) Differential abundance of phages infecting specific bacterial hosts in intestinal segments where significant changes were detected. The heatmap shows the log-fold change in phage abundance across treatment groups compared to the Blank control. (D) Bar plot of the total number of AMGs identified, categorized by their primary functional role. (E) Differential abundance of AMGs in the foregut and hindgut of AA and LY chickens. The heatmap displays the median difference in AMG abundance for treatment groups showing significant differences compared to the Blank control. PS: *P < 0.05, **P < 0.01, ***P < 0.001.

In the foregut of both chicken breeds, MCE treatments, particularly at medium concentration (MCE-M), consistently resulted in significantly higher richness (ACE, Chao1) and diversity (Shannon) compared to the CTC group. In AA chickens, only MCE-M significantly increased richness indices above the untreated control, while CTC supplementation significantly reduced community evenness (Simpson index). In the hindgut, responses were more variable and breed-specific, with MCE-M reducing estimated richness (Chao1) in AA chickens and MCE-L decreasing observed richness in LY chickens. Our findings demonstrate that CTC and MCE exert fundamentally distinct influences on the gut virobiota, moving beyond a purely community-level view to reveal host-specific and molecular consequences. At the ecological level, MCE, especially at medium concentration, acted as an ecological stabilizer in the metabolically active foregut, fostering a richer and more diverse phage community than CTC. This observation is consistent with the hypothesis that MCE promotes a more diverse and beneficial bacteriome, thereby expanding the host ecological niche space for phages. In contrast, CTC's reduction of community evenness signifies a selective pressure that favors a few dominant, pre-adapted phage taxa, likely through the reduction of bacterial diversity and niche availability.

We next analyzed phage abundance based on their predicted bacterial hosts. In the foregut, the abundances of phages infecting *Escherichia, Enterococcus* (and its relatives *Enterococcus_F, Enterococcus_E, Enterococcus_B*), and *Ligilactobacillus* were significantly altered (Fig. 2C). Phages associated with *Escherichia* and *Enterococcus* decreased in the CTC and MCE-L groups but were stable in MCE-M/H groups. Conversely, *Ligilactobacillus* phages increased in abundance in the CTC group. In the hindgut, phages targeting *Desulfovibrio* were significantly reduced specifically in the MCE-L group. This principle of host-dependent effects is further crystallized by examining specific phage-host pairs. The concurrent suppression of *Escherichia* and *Enterococcus* phages by CTC and MCE-L suggests a disruption of a potential pathogenic synergy between these genera. The stability of these populations under MCE-M/H, however, points to a more nuanced, "top-down" ecological management that suppresses virulence without collapsing the underlying network. The targeted reduction of *Desulfovibrio* phages (and by proxy, their host) in the hindgut by MCE-L is a positive signal, indicating an ability to mitigate a known contributor to gut dysbiosis even at low concentrations.

Functional profiling identified 43 AMGs within the vOTUs, involved in nucleotide, carbohydrate, amino acid, and vitamin B12 metabolism (Fig. 2D). The most prevalent AMG was a DNA methyltransferase (DNMT1). Differential analysis revealed that most AMGs were downregulated in MCE groups (Fig. 2E). Strikingly, the DNMT1 gene exhibited opposing regulation: it was downregulated in the foregut of MCE-treated AA chickens but upregulated in CTC-treated LY chickens, suggesting a compartmentalized and treatment-specific effect (Fig. 2E).

Functionally, these AMG abundance patterns point to a potential epigenetic dimension in the phage-additive interaction. The upregulation of DNMT1 in CTC-treated groups implies an enrichment for phage populations equipped to evade bacterial restriction-modification systems via molecular mimicry (Takahashi et al., 2025), thereby escalating the co-evolutionary arms race. Conversely, its downregulation under MCE treatment indicates a de-escalation of this conflict. We propose that by dampening overall bacterial virulence and activity, MCE lowers the selective pressure for sophisticated phage counter-defenses, thereby favoring stable coexistence over antagonism. The membrane-disrupting action of MCE's key alkaloid, sanguinarine (Qu et al., 2025), may synergize with this ecological effect by physically facilitating phage infection. Despite the lack of direct expression data, these distinct abundance patterns robustly highlight that MCE and antibiotics impose fundamentally different ecological selection pressures on the viral community. This virome-mediated homeostasis, characterized by preserved diversity and de-escalated antagonistic co-evolution, is consistent with the phenotypic improvements previously recorded in the same cohort, where MCE supplementation significantly enhanced body weight gain and feed conversion ratios (Huang et al., 2018). This concordance suggests that the viral ecological stability identified here is a key mechanistic underpinning of the zootechnical benefits observed in phytogenic-fed poultry.

We also acknowledge several limitations in this study. First, as mentioned above, this is a bioinformatics analysis based on metagenomic DNA. Therefore, our findings regarding AMG functions and phage-host interactions are predictions that reflect genetic potential rather than active gene expression, and need to be verified by future experiments (such as phage isolation and infection assays). Second, viral abundance was quantified via metagenomic read mapping, which detects viral DNA but does not distinguish between infectious virions and free DNA or inactive particles. Additionally, despite the use of state-of-the-art tools, host prediction remains a challenge in viral ecology, potentially influencing the precision of the inferred association networks. Finally, while our dataset encompassed key developmental stages and two commercial breeds, future studies incorporating broader genetic backgrounds and diverse rearing environments are needed to verify the generalizability of these spatiotemporal patterns.

In summary, this study systematically characterizes the spatiotemporal developmental landscape of the broiler gut phageome and reveals that phytogenics (MCE) and antibiotics (CTC) drive divergent ecological trajectories within this viral community. Unlike antibiotics, which impose selective pressure that reduces community evenness, MCE fosters a more diverse and stable virobiota, likely by shaping a healthier bacterial host environment. These findings offer a novel, viro‑centric perspective on the microecological effects of antibiotic alternatives, suggesting that phytogenics contribute to gut homeostasis by modulating interactions from the ecological down to the potential epigenetic level.

## Ethics statement

This study was conducted exclusively using publicly available metagenomic data downloaded from the NCBI Sequence Read Archive (SRA) under BioProject accession number PRJNA417359. As this research involved only the bioinformatic analysis of pre-existing datasets and did not involve any direct handling, intervention, or euthanasia of live animals by the authors, specific Institutional Animal Care and Use Committee (IACUC) approval or descriptions of euthanasia methods are not applicable to this study. The original animal experiments were performed in accordance with the ethical guidelines as described in the primary publication associated with the dataset.

## Funding

This study was financially supported by the National Key R&D Program of China 2023YFD1300300, the China Agriculture Research System of MOF and MARA (CARS-42-2) awarded to XW. Deutsche Forschungsgemeinschaft (DFG, German Research Foundation - Emmy Noether program, Project No. 273124240; and SPP2330, Project No. 464797012), and the European Research Council Starting grant (ERC StG 803077) awarded to LD.

## CRediT authorship contribution statement

**Li Yang:** Writing – original draft, Visualization, Formal analysis, Data curation. **Jinlong Ru:** Writing – review & editing, Project administration, Formal analysis. **Shun Guo:** Writing – review & editing, Methodology. **Xueqin Yang:** Writing – review & editing, Methodology. **Pengying Li:** Writing – review & editing, Methodology. **Li Deng:** Writing – review & editing, Funding acquisition. **Xia Wang:** Writing – review & editing, Supervision, Project administration, Methodology, Funding acquisition, Conceptualization.

## Disclosures

The authors declare that they have no known competing financial interests or personal relationships that could have appeared to influence the work reported in this paper.
